# For a Psychosocial Approach to Decent Work

**DOI:** 10.3389/fpsyg.2016.00422

**Published:** 2016-03-31

**Authors:** Jacques Pouyaud

**Affiliations:** Laboratory of Psychology (EA 4139), University of BordeauxBordeaux, France

**Keywords:** decent work, activity, career counseling, professional stance

## Abstract

The notion of decent work was developed by the International Labour Organization 20 years ago. The notion is now well known by lawyers, economists, and sociologists, and even if it appears difficult to define it clearly, it constitutes a useful general framework with which to think of the relationships between policy practices, work market globalization, and human rights principles. The fields of career guidance and counseling psychology are highly concerned by questions of social justice and human rights that people experience through work. Career choices being made in a liquid and unstable society, incompatibility between individualist values and collective work issues, increasing psychological health problems at work, work-family balance in precarious job situations, the growing necessity of mobility, adaptability or flexibility… all of these questions are at the heart of current career counseling practices, and concern the decent work debate. Nevertheless, the notion of decent work is not well developed in the field of vocational psychology. Despite its relevance, it is difficult to operationalize the policy and human rights principles during career counseling sessions. The article aims to explore the usefulness of the concept for career counselors, and to propose a psychosocial framework that incorporates decent work in career counseling practices. The first part of this article presents the theoretical bases of the notion of decent work and their possible use in the field of psychology. It deals with the necessity of a multilevel and psychosocial perspective, that takes into account both objective and subjective dimensions of decent work. The second part focuses on a case study illustrating how the notion of decent work emerges during counseling sessions. Four levels of the work experience linked with subjective and objective dimensions of decent work are explored; the personal level, the activity level, the collective level, and the social level. Finally, the third part discusses and proposes a framework based on the analysis of the four dimensions of work (activity, trade, others and society) in order to integrate a psychosocial view of decent work into career counseling.

## Introduction

At first glance, the notion of “decent work” is not a psychological concept. It was first defined by the International Labour Organization (ILO) in 1999, and finds its roots in fundamental moral principles of justice and equality for human rights. The utility of the notion lies primarily in its ability to support and enhance political actions for sustainable development. Thus, multiple publications already explore decent work in the fields of policy, economics, law, and even philosophy, but psychology remains mostly absent from the debate around decent work.

Nevertheless, decent work has recently begun to find a foothold in the field of career counseling psychology with the input of authors like J. Guichard and D. Blustein. For the former, we will refer to his conceptual work about self-construction (Guichard, 2009; Collin and Guichard, 2011), his association with the Life design constructivist perspective (Savickas et al., 2009), and his leadership of the Unesco Chair ‘Lifelong Career Guidance and Counseling’ where he encourages career practitioners and researchers to expand their concerns to more political dimensions (Guichard, 2015). For the latter, we shall refer to his theoretical perspective the “psychology of working” (Blustein, 2006, 2013), which provides a useful framework for the notion of decent work to be effective.

However, in order to use this concept in career counseling practice, it is still necessary to redefine or clarify it from a psychological perspective. Indeed, although the ILO definition explains the “objective” economical and moral conditions that make work decent, it does not explore more subjective aspects. The definition is therefore incomplete for a psychologist because of its lack of a subjective dimension. The core question is how the subjective meaning of work can be a part of the conditions that make work more or less decent for the individual *and* for society.

Several authors have already pointed out the imprecision of the initial ILO proposition and since 1999, conceptual efforts have been made to operationalize the concept (e.g., Deranty and MacMillan, 2012; Burchell et al., 2014; Di Ruggiero et al., 2015; Sehnbruch et al., 2015). The present article is based on these various propositions in order to formulate a psychosocial conceptualization of decent work, and to integrate the political aims inherent to decent work into career counseling practices.

The first part of this paper presents the theoretical bases of decent work and their possible use in the field of psychology. The second part focuses on a case study illustrating how the notion of decent work emerges during counseling sessions. The article will conclude with a discussion of concepts and methodologies to position a psychosocial view with the aim of integrating decent work into the career counseling field.

### Research aims

This study and the research it is based on aim to explore the usefulness of the notion of decent work for career counseling theorists, researchers, and practitioners. Moreover, it aims to propose a psychosocial framework for incorporating the decent work perspective into career counseling theory, research and practice.

### Theoretical question

#### From economical policy to psychology

As an answer to the global economical crisis of the beginning of the 90's, the ILO “made ‘decent work’ the centerpiece of its recommended recovery strategy” (Bisom-Rapp, 2011, pp. 1–2). But what is decent work? Presented in 1999 by the director of ILO Juan Somavia, decent work “involves opportunities for work that is productive and delivers a fair income, security in the workplace and social protection for families, better prospects for personal development and social integration, freedom for people to express their concerns, organize, and participate in the decisions that affect their lives and equality of opportunity and treatment for all women and men” (http://www.ilo.org/). Deranty and MacMillan (2012) expound the history of the ILO initiative and explain how the definition lies within the ILO principles created in 1919, upon a tripartite foundation: governments, employers and unions. The objective was then to participate in social justice through better organization of employment. The partners need to agree on common standards to regulate the labor market with logic other than that of the market economy. This first initiative was then reaffirmed, actualized, and specified three times after this date: in 1944 in the “Declaration of Philadelphia,” in 1998 in the “Declaration on Fundamental Principles and Rights at Work,” linked with the Human Rights Principles (United Nations Declaration of Human Rights, 1948) and in 2008 in the “Declaration on Social Justice for a Fair Globalization.” The authors summarize these principles: “[first] labor is not a commodity [Second…] it is not enough for a person to be employed; rather their employment must also afford them the opportunity to fully express their skill and in so doing contribute to the common well-being to the greatest extent possible [Third, this means defining core labor standards which are…] freedom of association and effective recognition of the right to collective bargaining; the elimination of all forms of forced or compulsory labor; the effective abolition of child labor; and the elimination of discrimination in respect to employment and occupation” (pp. 388–389).

Finally, in 2008, four pillars of decency defined in relation to human rights were decided to constitute the ILO framework for a sustainable economic recovery: “employment promotion, social protection, social dialog, and fundamental rights” (Bisom-Rapp, 2011, p. 2).

This short historical overview clearly locates the notion of decent work in the fields of economics, law, and politics. It primarily stresses the importance of human values for the organization of society and the working world, but this approach has also been criticized for its imprecision and its difficulty to be operationalized. Burchell et al. (2014) and Sehnbruch et al. (2015) for example, underline the terminological confusion that occurs sometimes in publications between words like “job,” “work,” “employment,” “job quality,” and “quality of work.” This confusion makes difficult the construction of clear and common indicators of what constitutes decent work. Furthermore, the lack of clear indicators makes international comparisons difficult, which explains the relatively low impact of the decent work proposition on policy, academic education, and research. The authors compare the decent work initiative to the “Human Development” or “Capacity” Approach (Sen, 1999, 2010; Nussbaum, 2000) which is, on the contrary, well operationalized (through the HDI–Human Development Index) and which, therefore, allows international comparisons and direct applications in policy decisions. The ideological roots of the concept, its aspirational dimension (Peccoud, 2003), and its grandeur seem to be a major constraint to its development and use. From a law perspective, this constraint is also clearly associated with the difficulty in establishing the legal and social responsibility of internationalized corporations (Javillier, 2007).

The main difficulty in elaborating a reliable measure of decent work is that it would needed to take into account multiple levels of analysis simultaneously. Ensconced in Urie Bronfenbrenner's theoretical model of Ecology of Human Development (Bronfenbrenner, 2005), a contextual and embedded view of decent work can be defined from the micro system (the actions and the relations between the individual and his/her actions), to the macro system (culture, religion, world of work, values…), passing through the meso and exo systems (groups in which individual interacts, and groups in which these groups are embedded). Without such a holistic perspective taking into account the various contexts of life, it is indeed difficult to understand how more or less decent work can be subjectively experienced by individuals.

From a legal studies perspective, MacNaughton and Frey (2011) show how decent work can be positively enhanced by the addition of a “holistic human rights approach.” With the same logic, and in order to use the concept in psychology, a subjective definition of decent work must be added to the objective one, highlighting how interactions (between individuals in systemically organized embedded contexts) construct the conditions for equality and individual capacity to develop. (Burchell et al., 2014) underline that the capacity approach developed by Sen (1999) and elaborated by Nussbaum (2000) allows a method of addressing this question of subjective definition of work through the notion of “adaptative preferences.” The capacity approach is characterized by “the ability of people to adapt to unfavorable circumstances (including poor employment conditions), which distorts their ability to evaluate their job characteristics objectively […] Because expectations vary considerably between countries, it is often the case that a developed country has lower aggregate job satisfaction than does a developing country. The same process can explain why some less advantaged groups of workers (e.g., women) have higher satisfaction levels than workers with objectively better working conditions” (p. 465). These two perspectives—decent work and capacity—appear to be more complementary to one another than opposing allowing the construction of a psychosocial view of the question. Decent work focuses on ideological conditions of work, while the capacity approach deals with the possibility inherent in the conditions of the individual's capacity and freedom.

#### Career counseling and decent work: Defining a strategic stance

The previous two approaches, both of which come from the economic world, can resonate with a social, organizational, or career counseling psychologist, especially if this practitioner sees his/her work as an ethical and political one (Guichard, 2003). If, as a psychologist, one's core activity is to help individuals construct and develop a capacity for action in relation to others and for the well being of the society, then the professional stance is to serve, at the same time, as an agent for socialization and individualization.

From the theoretical perspective of “active socialization” (Curie, 2000), “the process of socialization and of individualization aren't opposite, they are supporting each other” (p. 202, free translation). The strategic stance involves helping individuals realize a work to “interlace,” on the one hand, society and institutions that are vectors of conflict and alienation, and on the other hand a personal history, which is the integrated story of an individual's fight against external constraints in an effort to expound a personal world in which one wants to live. The result is a more or less decent environment that we individually and collectively build according to our freedom of choice and social latitude.

For the career counseling psychologist, assisting this interlaced construction often means adopting a person-centered approach and an “unconditional valorization” of the individual (Rogers and Kinget, 1962, cited by Curie, 2000, p. 202), but it also draws on “an action of mediation of the individual in cooperation with others” (p. 203). In other words, this can be described as a co-construction of projects (Young et al., 1997) on the micro and meso systemic level.

More generally, this professional stance meets the aims of the critical psychology perspective (Teo, 2005; Fox and Prilleltensky, 2009; Stead and Perry, 2012) and the social role that psychology should play for fair and sustainable development. A holistic, constructivist, and ecologic approach to decent work that can be used in psychology may contribute to this mission. As pointed out by Prilleltensky and Prilleltensky (2006), the well being of a society depends on the well being of the individual, but conversely, the well being of the individual also depends on the well being of society. To define the strategic stance, Prilleltensky and Stead (2011), describe the client-counselor relation as a dilemma between adjustment and confrontation with the environment (society, work, institutions). This “Adjust-Challenge Dilemma” not only regulates the capacity of the counselor to participate in the development of the general question of well being (both for the individual and the society), but is also the key for action.

The career counselor who tries to support the individuals in developing their own well being through self-construction in relation to others and others' well being, acts as an agent of active socialization. In doing so, he takes a political part, an ethical stance centered on the processes of personalization. He thus helps individuals to “permanently recreate a space of freedom that allows them to signify to themselves and to others that they are not the ones that the institutions would like to confine them” (Curie, 2000, p. 204, free translation).

The main objective of career counseling for the development of decent work is then the creation of a space of personal and social freedom, taking into account “the social and subjective conditions through which a subject, as a person, during a whole lifetime, and with the help of others […] objectifies and realizes the development of the potentialities that the personal history and the structures of socialization have let him/her glimpse (or have prevented)” (Beaumartin et al., 2010, p. 49, free translation).

#### A multilevel but fragmented perspective on work

To the question “what is good work or quality work?” psychologists already have some answers from a large corpus of work that has emerged since the beginning of organizational and career guidance, and counseling psychology. The primary part of this corpus defines the factors (personal, cultural, socio economics, organizational) that constitute the main predictors of well being at work. Nevertheless, this research has sometimes forgone the subjective aspects of work. The second part of the corpus is centered on the explanation of what the work means for the individual, but this research neglects to take into account more political and social issues. Again, it is difficult to forge a multilevel analysis of what constitutes a good job. Three examples are presented now as short illustrations.

For the first example, a large body of scientific literature is based on studies of occupational health, quality of life, quality of work, stress, and psychosocial risks at work. Gollac and Bordier (2011) propose a good summary of the groups of factors that play a role in defining psychosocial risks at work. Six groups are described: (1) *Work intensity and working time* (complexity, duration, work-life balance); (2) *Emotional demands* (relationships to clients, emotional inhibitions, fear, relationships to stressful situations); (3) *Autonomy at work* (autonomy within tasks, monotonous tasks, unpredictability, possibility of using competencies, pleasure at work); (4) *Social relations at work* (integration, socialization, justice, procedural justice, recognition, relations with colleagues, with hierarchy, wage, link between task and individual, work evaluation, social valorization, attention paid to well being, violence at work); (5) *Conflicts of values* (ethical conflicts, prevented quality, useless work); (6) *Insecurity* (job, wage, career security, risks of changes at work). Using this classification system, decent work will be work for which conditions are decent regarding well being and health risks for the worker. Bennett et al. (2003) coalesce organizational health perspectives into three streams of practice and research focused, respectively, on a healthy workplace, practice-oriented and consultative models that promote organizational health, and comprehensive multi factor health promotion and disease management programs. The main problem the authors highlight is that “although the different streams speak to a comprehensive view of organizational wellness, they tend to emphasize different levels of health (individual, job, or organizational) [they suggest] that occupational health psychologists must play a more proactive role in assessing relationships and integrating strategies among these levels” (p. 70).

On the individual side, a very broad field of study on the meaning of work, work satisfaction, work commitment, performance, and motivation, provides a complementary view. As a recent example, Mercure and Vultur (2010) identify “work ethoses”, that represent various relationships to work. Although, this approach is mainly sociological, based on the works of Bourdieu (1984), ethoses can refer to personal differences in work meaning, work values, and work engagement. Work ethoses are linked with life ethoses and are defined through three dimensions: centrality of work (and work-life balance), finality of work, and work engagement in relation to the dominant social and managerial norms. Six ethoses are described: *Autarky* (search for financial independence, autonomy); *professionalism* (search for personal development, valorization of competencies, peer recognition); *utilitarism* (search for personal satisfaction, consumerism); *egotist* (search for assertiveness through work and other life domains); *resignation* (work lived as a constraint); and *harmony* (search for consistency with personal values). These ethoses can be seen as personal translations of work experiences and work conditions. Objective decent work experiences defined through work conditions can be colored with various degrees of personal acceptance. Decent work is then work “lived as” decent through the filter of work ethoses. The authors also highlight that some ethoses (e.g., professionalism and egotist) are highly compatible with contemporary liberal management, which helps to internalize the core priorities of the new liberal spirit: valorization of the centrality of work, flexibility and mobility, and valorization of subjective engagement at work.

In the field of career counseling, the now classical approach of “social cognitive career theory” (Lent et al., 2000) provides an understanding of how individuals can create a personal perspective and experience work conditions to act and transform their environment. The theory is an application of the works of Bandura (1986), especially the concepts of self-efficacy and agency, on career issues. Work performance and career attitudes are results of “self-efficacy beliefs,” built from the interaction between the individual and a more or less afforded environment. Again, decent work can be seen as the personal meaning that individuals grant to their capability to regulate conflicts with work. Other theoretical constructs such as career adaptability (Savickas and Porfeli, 2012) and intrapreneurial self-capital (Di Fabio, 2014) are gaining attention in the field today, but again the main point of difficulty is the focus on the individual that doesn't take into account ethical and political levels of responsibility. The risk here is a reduction of ethical and political intentions and responsibilities, because of the possible fluctuations of the perception of work decency between individuals.

## Case study

The following case study illustrates how the notion of decent work appears in career counseling situations, from the individual level to more collective dimensions. It will later support a discussion of and proposition for a psychosocial perspective of decent work.

### Client background

Mrs. V is a 48 year-old woman who has been a social worker for about 26 years. Mrs. V says she always wanted to have a job helping others. During high school, she was already thinking of becoming a social worker. After she completed high school, she entered university, and moved away from her family. She then experienced several periods of depression because of loneliness. She completed her social worker diploma at the age of 25, and began working in an official institution. She got married and had two children. At 35, she divorced and 2 years later began a new relationship with another person. Her companion became ill and died 3 years later. During his illness, she began working part time in order to take care of him. Since then her profession has became harder to perform. One year ago, doctors diagnosed her with a serious disease, at which point she began to experience depression again. The occupational medicine department offered her a part time job. She has started thinking she would like to change jobs and do something else, because she can't stand her work anymore. Consequently, the department proposed for her to start a “bilan de competence” (see below for the detail). She met with a counselor at the elicitation center (“Centre Interinstitutionnel de Bilan de Compétence”) in order to find new professional prospects. At the beginning of the Bilan, Mrs. V said that she couldn't work anymore because she felt tired and powerless.

### Counseling process

The “bilan de competence” in France is a system of career guidance and counseling based on collaboration between social partners and state authorities. Since 1991, a law has established the right to attend a bilan and the way it must be organized. This program was proposed first for adults faced with the termination of employment and women wishing to return to work, then extended to young people without qualifications and to unemployed adults. Today, the bilan is available to all workers. Within companies, all employees with 5 years of experience, including at least 12 months in their present company, can request a leave of absence to draw up a “bilan.” “The bilan is intended to determine the current state of the individual's competence, and personal and occupational skills. The bilan is based on an assessment which may include a variety of methods (interviews, diagnostic assessments, self-assessment, tests, etc.) in order to draw up an occupational plan and, if appropriate, a training plan. It is the property of the person concerned and may not be communicated to a third party without the permission of the subject” (Perker and Ward, 1996, p. 137). This personal and occupational bilan is organized in three phases: the preliminary phase (defining needs, setting up a working alliance and information), the investigation phase (exploring and/or assessing values, interests, aspirations, motivation, general and occupational knowledge, skills and experiences), and the concluding phase (drawing up a summary that is a personal and confidential document only dedicated to the person). Only public or private organizations external to the company have the right to offer this service, following the rules defined by law: reliable methods and empirically-based instruments for assessment; qualified counselors; and confidentiality (the results of the bilan are the property of the individual and not of the company or of the funding organization). In 2007, 194,000 persons underwent a bilan in one of the 1100 approved centers (Dares, 2009). The main association that provides this service in France the Centre Interinstitutionnel de Bilan de Compétence (CIBC), which has 330 centers. Workers can access a bilan at their own request, at the request of their employer, or through employment services (in case of a training project for example). It is most often financed by the training budget of the company, or from the companies' compulsory contribution. In the case of unemployed and young people, the state or the region pays for the service.

In the case of Mrs. V, the company proposed to finance her bilan in order to help her recover and find a more protective working environment or new training. Her bilan lasted 2 months. It consisted of 10 interviews of 2–3 h each, all with the same counselor. The bilan followed the three phases: analysis of needs, investigation, and writing of a “summary document.” Like many bilan, even if the process is initially focused on work-related issues, it addresses health problems at work, as well as more global dimensions of people's lives. When Mrs V. came to the CIBC, she was asked to participate in research on career counseling practices.

### Research procedure

The case of Mrs V. is an illustration taken from a project conducted on the effect of counseling sessions in France (bilan de compétences). It is part of a larger research agenda, designed to study the effect of the dialogical and dynamic relationship between clients and counselors. The design was inspired by grounded theory (Fassinger, 2005), triangulation of perspectives in action research (Kidd and Kral, 2005; Polkinghorne, 2005), and self-confrontation procedure (Young et al., 1994). The research used multiple participants to make various analysis of the bilan in order to highlight the dynamic and developmental processes during counseling sessions. As pointed out by Polkinghorne, “by comparing and contrasting these perspectives, researchers are able to notice the essential aspects that appear across the sources and to recognize variations in how the experience appears. In this sense, multiple participants serve as a kind of triangulation on the experience, locating its core meaning by approaching it through different accounts. […] The use of multiple participants serves to deepen the understanding of the investigated experience ≫ (2005, p. 140). The research procedure, which can be described as Participatory Action Research (Kidd and Kral, 2005), was organized in 6 parts:

–Over 1–3 months, the 10 interviews of the bilan de competence are performed by a counselor from the CIBC. The interviews are videotaped. A first descriptive thematic analysis (Bardin, 1977) is conducted with advanced students in psychology in order to define the key moments and the dynamic structure of the bilan.
–One month after the bilan, a collective discussion session is organized between the students and the counselor. The discussion is recorded and provides an opportunity for the researcher to gain a second point of view on the key moments and the dynamic of the bilan. This session also allows the counselor to reconsider her process with the help of the students and their observations. The novice questioning of the students turns this session into a kind of “collective supervision,” in the sense that the counselor can rediscover her practice in the eyes of beginners. The objective of this session for the researcher is to validate the key moments marked previously, in order to determine the impact of each one for future work with the client.
3. Two months after the bilan, a career construction interview (Savickas, 2011) is conducted with the client. The interview is recorded and analyzed by the researcher in order to verify the choice of key moments. The objective is to have a third perspective on the key moments, and to analyze them in the light of the subjective career and life themes.
4. The researcher puts together a video with the key moments from the bilan. These key moments are the result of the triangulated perspective and analyses of the counselor, the client, and the students, as well as the researcher's analyses of the career construction interview. These key moments are turning points, in which the development of the relationship and the progress of the counseling take place (moments of unveiling, resistance, transference, etc.). The video is then viewed during a “self-confrontation session” (close to the interpersonal process recall procedure: Watson and Rennie, 1994; Larsen et al., 2008) between the counselor and the client, led by the researcher. The researcher helps the client and the counselor to jointly analyze what is occurring in the key moments, understood as instances of development. The self-confrontation session is also videotaped for an analysis of the change process.
5. At the end of the process, the counselor and researcher together choose some key moments of the assessment that will serve as an illustration to use with the CIBC team. A second video of key moments is made for this purpose. It may be used either collectively at a team meeting, or during another self-confrontation session with two colleagues wishing to pursue the process with one of their clients.

### Ethics

In this case, the research didn't need to be formally approved by an ethic committee for two main reasons. One, institutional ethical clearance is not required for interview studies. Two, the article presents a case study based on a counseling encounter as an illustration. Thus, the work referenced with the participant in the article was not research *per se*, but it was an intervention that was used to inform the article, from a research action perspective. Nevertheless, the research procedure followed the ethical standards of the latest version of the Declaration of Helsinki revised in Fortaleza (World, Medical Association [WMA], 2013), and of the French society of Psychology Ethical Guidelines (French Psychological Association, 2012). Prior to the interview, the research procedures were explained and the participants agreed to participate in the study by signing the informed consent. All participants were informed about their rights to withdraw from the study at any time.

### Data analysis

In order to analyze how the notion of decent work appears during Mrs V.'s bilan, we have used the thematic analysis (Bardin, 1977) performed by the supervised students during phase 1. The present analysis consists in bringing together the thematic analyses and a psychosocial model of decent work.

## Results

At the beginning, Mrs. V said that she could no longer work as a social worker because she felt exhausted and couldn't find the necessary strength to accompany people anymore. In addition, she thinks her work has evolved to no longer be appropriate for her. She explains that her institution does not allow her to give fair and equitable support to people in difficulty. For example, she was very upset when she saw that electricity was cut off to people in need who could not pay their bills.

According to her, working conditions have deteriorated over the years, and are now particularly degraded, with less autonomy, much more oversight, and a loss of team work and of collaboration with colleagues. The result is a more individualistic work environment in which she has to face the misery alone. For her, struggling with poverty is increasingly hard to bear. Every day she experiences the failure of bringing any real help. Ultimately, she thinks she is doing her job badly. But when she looks back at her career, she wonders if she really wanted to do this job, even at the beginning. She mainly describes the circumstances that guided her, but now that the demands of work have become more difficult she wonders if she is really made for that field. She has just emerged from a very difficult period during which her husband died. She is now alone with her two children who are almost adults.

She says the job has become less and less recognized and respected, even by the beneficiaries who only “use” her services in a “technical” way (i.e., to search for financial support) without engaging in a true helping relationship. She feels discouraged and tired of trying to help people in trouble without any means or recognition.

She asked the following questions to the counselor: Why does this occupation exhaust me so much? And how can I find another occupation that fits my values more? A thematic analysis shows five main themes that the counselor and Mrs. V discussed during the course of the bilan. The themes are illustrated below with direct quotes from Mrs. V (extracted from the first three meetings).

*Theme 1: Find the energy to re-engage in a project*.

“Today I have the impression that, in fact, there is no creativity at all. There is no surprise, and a certain weariness. It's actually so confining that it leaves little space to open new things, and to start projects” (Interview 1)

“The counseling will be successful if I can get involved in a project with motivation and not by default, if I have enough energy to get involved” (Interview 1)

*Theme 2: Allow myself to “remove barriers”–in relation to the institutional framework, to social norms, to the expectations of the environment, what is correct or not correct, and not be afraid to say what I really want to do*.

“For me the bilan will be successful if I can break down barriers, fears and have faith in myself, and it will help me to know what I want to do, and it will help me to give myself the means” (Interview 1)

“I really need to be able to say what I want, not staying in…because again I…I have some barriers…I do not dare.…I really need to dare to say what I want because…at the moment I tell myself that it's not possible, or that I am not able to, or that it will not allow me to earn a living” (Interview 1)

Theme 3: Social worker, why is it a job that no longer suits me?

“There is no more interest, no more interest to work as a social worker,…how the procedures currently replace…when I started in this profession, it was a profession that involved much more relationships and much less proceedings…Now that I am a social worker, there are lot of procedures, and we live in an increasingly restrictive society…so we are no longer helping people, we have to put people in boxes, and that's not why I chose this job” (Interview 1)

*Theme 4: Human relationships are at the heart of my job dissatisfaction—along with isolation and loss of collaboration*.

“When you personally meet with the people and witness all the suffering just because, I think there is such a degradation of the social fabric, that the situations are more and more heavy, and one's shoulders must be a bit strong…, I feel less and less capable to feel empathy. People quickly irritate me” (Interview 1)

“It's actually a big question for me…for example, in human relationships, the relationship…the human approach…all that, it has always been of great importance to me, be it for commercial purposes etc…it was the human relationship. These days…it's a bit ambivalent. I'm tired, and it's a kind of what I was saying previously…listening to people, it's really a mental strain…” (Interview 2)

Theme 5: Communicate and work with team

“I know that I'm a bit…like that. I sympathize, I have my colleagues, I sympathize…that's not a problem but I'm pretty individualistic in my way of working” (Interview 3)

“I mean if it's recognized, that I'm able to work with partners, well with partners…with colleagues…it's also a way to be recognized. Compared to my hierarchy…that will allow me to have a place in relation to my skills, that I do not know how to sell…My skills I make them on my own, in my corner.…I make them individually, in the moment…it's very interesting for me but it does not allow me professionally…I will not be able to work in a…and be recognized so that I can get a position in relation to that” (Interview 3)

Through these five themes, the counseling process with Mrs. V dealt with a gradual deterioration of her relationship to work and an increasing perception of her work as indecent. To her, the degradation seems connected to a personal fatigue (her recently painful life story, her midlife career situation, a need for change and a lack of positive perspective of evolution), and to the objective degradation of working conditions, in the institution, but also more generally in society. The “indecent dimension” of her work is thus related to the conjunction of subjective and objective relationships to work:

“That's what is difficult actually, to really see…is it the evolution of our profession that wore me out or is it personal? Because I think it's the two” (Interview 1)

More generally, four levels can finally be highlighted for defining the decency of work:

### Personal level: Life story

The work situation of Mrs. V resonates with personal fears that exist and persist in her life story. For example, the fear of not being able to be free and to express herself and the need (as a life theme) to be original, and to avoid to be constrained.

“My thought at that time was…I wanted to know…have more knowledge of what life is in order to help people and that is, not be restricted (…) I needed to see other things.” “This dissatisfaction that I still drag now, is a dissatisfaction growing old”

### The level of activity: (work as concrete action)

Gradually, the work of social worker has moved from an independent counseling relationship, to a relationship based on a search for social control. Mrs. V mainly expresses her loss of empowerment with a feeling of helplessness and worthlessness.

“My current role is a role of social control…control…it's no longer a helping relationship, it is a control relationship”

### Collective level: Interaction with peers

Mrs. V expresses that her work is more and more individualized. She also regrets the increasing difficulty to be recognized for the work she provides.

“I have trouble communicating with my colleagues…I am not the only one…well I mean it may be a state of mind…it's what I'm thinking, that maybe it's a general state of mind of our profession.” (Interview 3)

### Social level: Values

Finally, the evolving general mindset of society contributes to Mrs. V's subjective experience of her work devolving:

“In a society in crisis, I do not want to be Private Ryan anymore” (Interview 2)

The result of this multilevel degradation is an impassable situation—congested with fear, isolation and feelings of injustice—which leads Mrs. V to be unable to say what she wants. And for her, it is no longer possible to believe in her future.

## Discussion

### A psychosocial perspective on decent work

A strategic perspective on decent work in the field of career counseling must be rooted in a comprehensive, multilevel psychosocial analysis of work. Such a perspective certainly needs to consider work as a major life domain through which to explain self-construction and society. The question of the centrality of work in relation to other life domains is, then, of major importance.

The framework of the psychology of working (Blustein, 2006) clearly integrates this dimension. The theoretical perspective is based on Richardson's propositions (Richardson, 1993) that suggest enlarging the concept of career and opening research on work to pluralistic theoretical approaches such as constructivism and social-constructionism. Some core tenets constitute the basis of this framework (Blustein, 2013, pp. 7–9): It is necessary to use diverse epistemologies to understand the nature of working; Work is central in life and to mental well being, and has the ability to fulfill core human needs (survival, relatedness, and self-determination); Work is broader than career and includes “non paid work,” non work, working for survival, indecent work, and others because all these activities contribute to people's lived experiences in various contexts; Culture and relationship are core tools of meaning making for people's experiences; Understanding work means identifying how social, economic, and political forces influence the distribution of resources and affordances on a macro level. From Blustein's perspective, “there is considerable variability in individual's experiences regarding volition, and poor and working class individuals may experience little choice regarding their working lives […] ‘Working’ is thus a more universal and inclusive term than ‘Career”’ (Swanson, 2013). In considering working rather than career, this approach allows us to think of the relationships between concrete subjective experiences of work, and more social, organizational and political issues.

In the same way, the capability approach of Sen (1999), complementary to Bandura's agency, allows a multilevel conception of decent work. It helps understand how the micro and macro levels are linked together. As explained by Sen (1995), the capability approach involves “concentration on freedoms to achieve in general and the capabilities to function in particular” (1995, p. 266). The author distinguishes between functionings and capabilities. “A functioning is an achievement, whereas a capability is the ability to achieve. Functionings are, in a sense, more directly related to living conditions, since they are different aspects of living conditions (e.g., personal, social, and environmental characteristics). Capabilities, in contrast, are notions of freedom, in the positive sense: what real opportunities you have regarding the life you may lead” (Sen, 1987, p. 36).

It is between functionings and capabilities that a psychosocial definition of decent work can take place. The multilevel analysis that can support a psychosocial view of decent work can be described from the micro to the macro level:

“The capability approach to a person's advantage is concerned with evaluating it in terms of his or her actual ability to achieve various valuable functionings as a part of living. The corresponding approach to social advantage—for aggregative appraisal as well as for the choice of institutions and policy—takes the set of individual capabilities as constituting an indispensable and central part of the relevant informational base of such evaluation” (Sen, 1993, p. 30). Well-being is thus defined as people's capability to function (to work, to relate to others) and to freely undertake the actions and activities that they want to engage in, in order to be whom they want to be.

A psychosocial definition of decent work should consider work experience (in its relation to other life domains) as a central part of self-construction. The working experience is then a major dimension of an identity structure that can be described as a dynamic system of subjective identity forms (Guichard, 2009; Guichard et al., 2011; Guichard and Pouyaud, 2014). A subjective identity form (SIF) is a construction and implementation of oneself in a particular setting (a set of ways of being, acting, interacting, and thinking of oneself in a particular role). This does not mean that the professional role constitutes a more important SIF than the familial or social ones, but it means that the adjustments inside this system, between work and non work subjective identity forms, will contribute to work being perceived as more or less decent. In that sense, the working identity form plays a central role in self-construction as it helps to design and construct the self in other meaningful non-work subjective identity forms. Indecent work (work that doesn't respect the human rights principles) is then work that degrades the dynamism of the identity structure because of living, working or organizational conditions. As illustrated by Mrs. V's case, the four thematic levels of analysis highlight how her working subjective identity form as a social worker (in terms of personal history, professional action, values, and social role) is not allowing her to develop anymore.

This centrality of the working life is also well described by the French psychoanalyst Dejours for whom working is “a subjective activity that mobilizes the emotional, cognitive, and moral capacities of the individuals, and in return transforms them, for better or for worse” (Deranty and MacMillan, 2012, p. 395). For Dejours, work plays a “decisive role in the elaboration of the civil relationships whereby individuals can live and act together [this is…] the thesis of the political centrality of work” (Dejours, 2009, p. 14, free translation). The idea is close to Rawls' view about the meaning of work: “the lack of opportunity for meaningful work and occupation is destructive of citizen's self-respect” (1996, p. 159, cited by Deranty and MacMillan, 2012).

Deranty and MacMillan (2012) endorse Dejours' propositions for a more comprehensive view of decent work. The authors explain that “an important aspect in determining whether work is decent or not is constituted by the activity entailed in work or by the content of work” (pp. 402–403). “[…] the focus on the very activity of work in its unfolding, and on the impact of this unfolding on subjective capacities, provides crucial indications as to what can make work interesting, a vector of intelligence and initiative, and indeed a place of concrete democratic action” (p. 395).

Following their proposition, and as briefly illustrated by Mr. V's case, a multilevel analysis of the dynamic system of identity forms during counseling sessions allows an understanding of the psychosocial meaning of work. The following framework combines the previous theoretical stances to propose a guide to structure this multilevel exploration.

**Figure 1:**
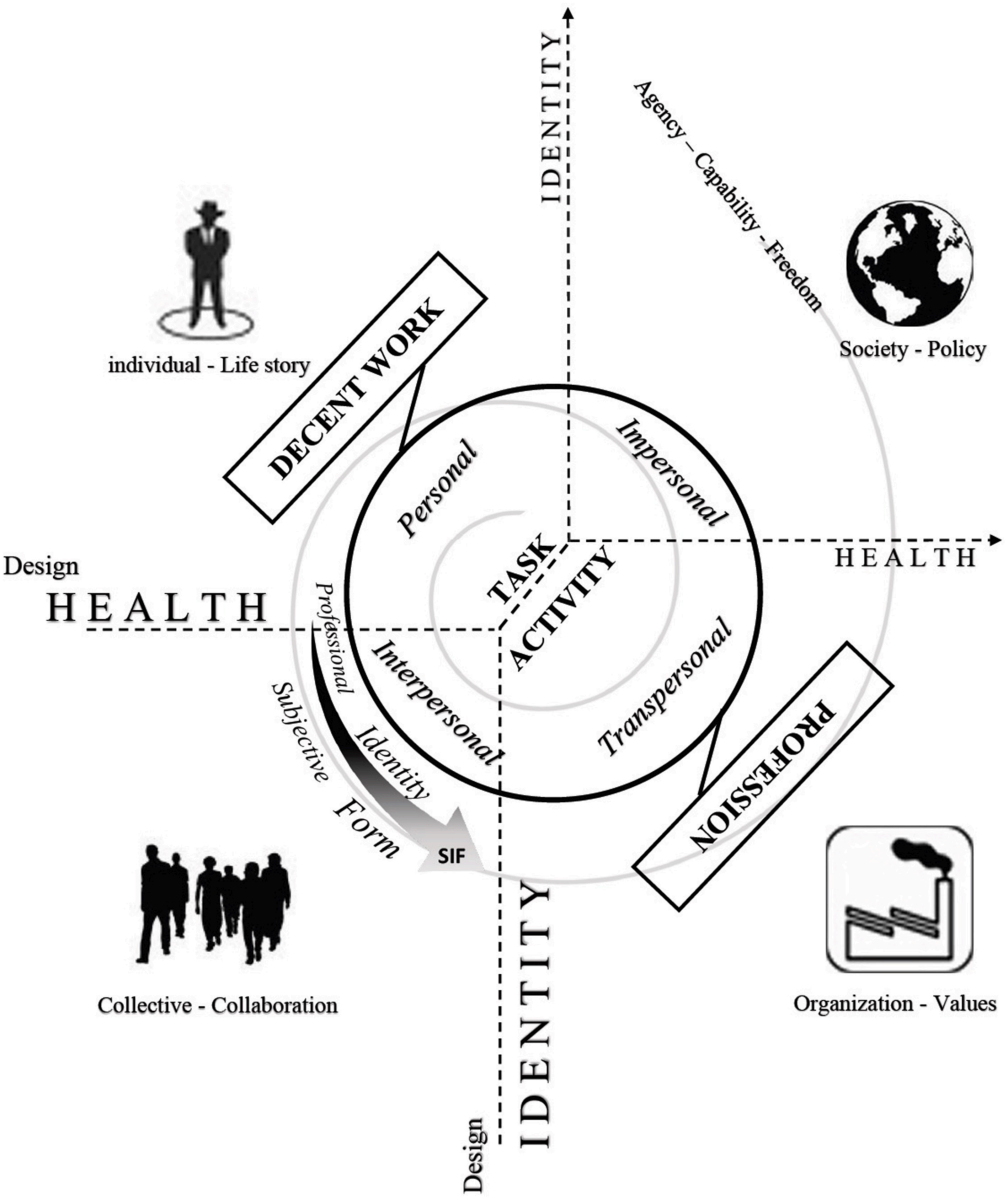
**Decent work as a psychosocial relationship between an individual and a context of production with others (collective, organization and society—work as a conflict to resolve)**.

This framework is illustrated in Figure 1 and is based on the following 5 proposals:

- Four levels of embedded contexts constitute the developmental environment for the individual (the workplace in connection with other living environments constitutes the interactional contexts where the system of subjective identity forms can occur).
- A spiral suggests possible development in this environment. Development is rooted in the conflict lived by individuals at work, between the task and the activity (as a response to the task). Within this response is potential: capability and personal agency, which depend on the freedom granted by the embedded contexts.
- The “psychosocial set” that allows the potential for development is the “trade” or “profession,” as defined in the theoretical framework of the “Activity Clinic” approach (Clot, 2009). It is constructed through four dimensions of relation to working: personal/intrapersonal/interpersonal/and transpersonal dimension.
- Decent work, viewed as the psychosocial conditions of well being, is a trade that draws the individual into a dynamic system of subjective identity forms supported by the four dimensional architecture of work (and mainly by the personal and intrapersonal ones, which link the psycho and the social).
- Finally, this set impacts health, well-being and identity both at the individual and collective levels.

The following sections explain more clearly theses proposals.

### Activity

The starting point corresponds to the micro level of action. Work is defined here as an activity and, more specifically, as a dialectic with the working environment. This theoretical view is the classical distinction made by French-speaking ergonomists between task and activity (Ombredane and Faverge, 1955). As pointed out by Clot, “Whereas international ergonomics focused on the engineering of task and artifacts, French-speaking ergonomics was organized around activity and health with the intention of preserving and developing the operators' power to act in the workplace” (2009, p. 286). The work situation demands that the individual answer to a prescription (a task) taking into account internal conditions (age, health, gender, intelligence, etc.) and external conditions (tools, peers, hierarchy, management, noise or temperature conditions, etc.). But the answer that the individuals are constructing within these conditions is always different from the expected task. This construction is called activity and the gap between the task and the activity is an indicator of what the individual subjectively adds in order to overcome the conflict. As depicted in Figure 1, this conflict is the starting point of individual and social development at work. For Mrs. V., this conflict is circumscribed, as the professional action is impossible for her to sustain.

The theoretical approach of Activity Clinic (Clot, 2009; Kloetzer et al., 2015) expands the ergonomic point of view, especially on the basis of Vygotsky's concepts. Two complementary analyses are made, one on the notion of activity, and the other on the notion of “Profession.”

For the first, Clot distinguishes what can be observed from the activity (gestures, actions, interactions, tools used) called “realized activity,” from what may have been done, but did not survive in the working conditions, called “the reality of the activity.”

As pointed out by Vygotsky, “behavior is a system of victorious reactions…at every moment, the individual is full of unrealized possibilities” (Vygotsky, 1999, pp. 266–267). The reality of the activity refers to “what is not done, what can't be done, what one tries to do without succeeding, the failures, what one would or could do, what one thinks or dreams to do elsewhere […] what is done to avoid to do what is to be done, or even what is done without the will to do it. Not to mention what has to be redone” (Clot, 1999, p. 119, free translation).

Activity is defined as a story of the development of action and of the subjective investment of the individual in order to “be” and to “do” beyond any conflict with the task. What can be observed from the activity is a trace, a result of this story. Helping people at work means co-constructing the resources of the development of their activity, and re-opening the possibilities for self-construction through work. For Clot, “the transformation of actions for developing the subjects' power to act is the object itself of a basic and field-based science of psychology” (2009, pp. 287–288). From a decent work perspective, Deranty and MacMillan express that “at this first level, decent work would be work in which the design of the job and the organization of work enable the individuals to make full use of their capacities in the deployment of their activity” (2012, p. 399).

### Profession and decent work

The second analysis on activity made by Clot helps to understand how this could be possible. The Activity Clinic approach extends the notion of activity by explaining how the individual level is dependent on more collective and social levels. The notion of “Profession” can be used to analyze the importance of others (e.g., professional collectives, peers, managers, institutions) in the development of activity, and in the enhancement of individual capacities at work. A profession is defined through four dimensions, which consolidate a subjective armature of work activity (see Figure 1). All dimensions can be places for work development, as well as for decent work development.

The first dimension is the “personal” one and refers to the first level described previously. It corresponds to “the specific way one is performing one's activity, according to one's specific skills, knowledge, history, life story, professional experience, preferences, moods, expectations, worries, goals, hopes, and desires” (Kloetzer et al., 2015, p. 61). The second dimension, called “interpersonal,” moves the analysis to the interaction zone. Indeed, activity exists and evolves within action and interaction with others. Personal action is always mediated by others who can support, restrain, or change its development. On the third level, called “transpersonal,” the activity becomes collective. The profession exists at this level as a “transpersonal memory” of shared activities. Historically built from the collective development of the professional milieu, this collective memory plays the role of a constraint (as an unofficial culture, like the rules to be followed in order to be recognized as a good professional by peers), and a resource because it helps resolve the common professional conflicts of work activities. This level also involves a “professional identity” built upon a common way of working in a particular profession, product of the collective history of professional activity. It refers to “the usual ways of acting and interacting, speaking, doing, and relating to people and things in a professional way that are established in a specific work environment” (Kloetzer et al., 2015, p. 61). As depicted in Figure 1, this can be defined as a Professionnal Subjective Identity form. This level is of major importance, because “it is a binding characteristic across generations and individuals, always at risk of disappearing if it is not reconstructed in the course of personal and interpersonal activities” (Kloetzer et al., 2015, p. 61). The personal and interpersonal activities (first and second dimensions) refer to the “professional style.” This is where the “professional identity” can develop by integrating the individual and interindividual creativity used to resolve the dialectic of working. It thus involves the processes of personalization. If the first two dimensions are places for novelty and innovation, the intrapersonal dimension is a first level of cooperation and solidarity at work and a first source for a personal recognition. It is the starting point for the employment promotion, social protection, social dialog, and fundamental rights that constitute the four pillars of decent work. For this reason, psychologists can find the link that allows them, in their own work, to contribute to the enhancement of decent work by working on the processes of personalization that reconstruct professional subjective identity forms. However, these efforts can't be effective without social and institutional recognition regarding more impersonal descriptions of the profession, which refer back to the task. Indeed, the fourth dimension defines the profession as “impersonal.” It corresponds to all the official characteristics of work organization (e.g., tasks, job profiles, career rules) that build work outside the individual level.

As pointed out by Kloetzer et al. “in this model, all four dimensions are bound together, but antagonisms may provoke a loosening of these bonds. The feeling of sharing the same experience at work may disappear due to interpersonal conflicts. A trade that is deprived of transpersonal mediation may degenerate into destructive opposition between a personal, solitary work exercise, and impersonal, spurious work injunctions from the organization, with all workers at risk of work depersonalization” (Kloetzer et al., 2015, p. 62). For example, in the case of Mrs. V., the way she perceives herself in terms of values or skills (which are parts of the description of subjective identity forms) is no longer supported by her professional environment. We may say that the professional scenarios no longer sustain her professional self.

Career counseling psychologists who want to integrate the decent work perspective into their practice could have an interest in understanding how the dynamic system of subjective identity forms of their clients integrates these four levels as resources or constraints for self-construction and personal and social recognition (see Figure 1).

In doing so, they could play a role in helping clients to engage in new “ecological transitions” (reflecting their relationships to peers, to task, to hierarchy and to institution). Nevertheless, such a practice will also needs another type of intervention that can help the professional milieu to be more inclusive in order to accept such ecological transitions, development and capacity to transform society.

### Others

Decent work exists within the relationship between a subjective meaning of work and an objective definition of what constitutes good working conditions. It withholds the structure of personal identity at work. But this structure also needs to be supported by others. Two levels underlined by Deranty et al. concern the relationships to others—work as cooperation (which can refer to the transpersonal dimension of work) and work as political culture (the impersonal dimension).

We have already outlined that the collective of peers could be helpful for individuals to perform tasks and resolve activity conflicts. The collective also has the power to recognize the quality of good work (recognize a “professional style” as a good one), and to support human rights at work. As noted by Dejours, the only true recognition of the quality of work is made by peers who can have a “judgment of beauty” about the work (Dejours, 2009, pp. 42–43). Decent work is then work that can be recognized by peers according to the conditions of certainty and cooperation. These two conditions are essential for the profession to be a mediating agent of personal and collective development alongside the institutional prescriptions. Career psychologists need to think of how they can contribute to engaging organizations and institutions to be more cooperative and positive places.

One main difficulty in career counseling is that career interviews are often “disconnected” from concrete work situations, and especially from their collective dimensions. The involvement of pairs in the co-construction of possible solutions and potential development is, however, of high importance. In the case of Mrs V., this certainly constitutes a core issue. Because she is already in conflict with her colleagues, and interviews are conducted outside her work, it can be difficult for the counselor to intervene further than personal dimension. One of the main challenges for the career counseling field is perhaps to extend the practical interventions to the collective and political levels inside the workplace.

The specific methodology used by the Activity Clinic (see Kloetzer et al., 2015, for an example) can provide a complementary approach to individual interviews. It consists of grounded research-action methodologies based on the idea that working is a process of learning and development. In building, for example, cross self-confrontation situations, the researchers elaborate a dialogical Zone of Proximal Development with workers that can support the development of the four-dimensional dynamic architecture of the profession (personal, interpersonal, transpersonal, and impersonal dimensions). Phases 4 and 5 of the participatory action research described previously explicitly refer to such perspectives, with the aim of developing the profession of counselor into the CIBC, and allowing the counselors to enhance their power to act (and for example help them to integrate the decent work concerns into practices). Because the main developmental tool within this methodology is professional controversy, cooperation and support are the first assumptions that must be sustained.

### Society

As depicted in Figure 1, yet another level of analysis needs to be developed. As noted by Dejours “the fact that the relationship between work and life turns into happiness or misfortune does not depend on individual economy. Success in the face of the personal challenges that constitute the subjective experience of work depends on the social conditions of work” (2009, pp. 42–43, free translation). The social conditions of work are part of the political culture. Everyone can see how the reality of the precarious and flexible job market makes solid self-construction difficult. Authors like Dejours have a severely pessimistic view of the impact of our organizational, economic and political culture on post-modern identity construction, and on the global capacity of our society to maintain democratic and human rights values. For Dejours, the organization of today's work makes malicious behaviors commonplace, and endangers democracy. The economic war, at the heart of the neoliberal system, has created a lot of wealth, but also a lot of inequality, injustice, unemployment, discrimination, marginalization, and precariousness (Piketty, 2013). The work conditions today are lead by fear (fear of unemployment, fear of the future, fear of climatic evolution, fear of communities, fear of exclusion). Economic health is determined by the stock market's confidence thermometer, like an aggregate of all individual fears. The result is suffering and malicious behavior that have become structural components of today's societies. When fear is accepted as a natural dimension of working conditions, work becomes a theater for the normalization of evil (where inequalities and inequities are perceived as natural). The result is, for Dejours, the development of attitudes of submission and domination at work, with these behaviors becoming reasonable, justified, and rational (as social norms). Every “post modern citizen” is therefore supposed to follow one of these two options and accept living in an indecent world, framed as an injunction to be realistic. Today's organization of work is the theater of a fight between domination (alienation) and democracy for one, and between individualism (isolation) and solidarity for another. Fear at work as a norm is the origin of isolation and of the loss of democracy, because it muzzles cooperation and collective action. On the other hand, work can also be seen as a major key to human development, if the cultural and organizational conditions allow work collectives to sustain personal identity. As pointed out by Deranty et al. “because it can be such a destabilizing experience for individual subjective identities, the very organization of the work process can be a major factor in entrenching general forms of social domination […] By contrast, because it can also be a major vehicle for the development of cognitive, emotional, and moral capacities, work can also be a vehicle for democratic life and respect of others” (Deranty and MacMillan, 2012, p. 401). The strategic stance of career practitioners should enable them to contribute to political and economic changes through the development of individuals and institutions in which they are included.

Finally, the following “formula” could be used as a first step in that direction:

Work is decent when it allows the individual and the society to be healthy, in the following ways:

- By consolidating social and individual identity to allow the realization of tasks
- By developing work activities, through recognition, evaluation, and confrontation with others (especially peers)
- By integrating concern for others into work (for more justice, equity and democracy).

## Conclusion

The notion of decent work is not easy to define from a psychological perspective. But from the point of view of career counseling, the notion clearly points out the importance of human rights in career issues and practices at work. Most of the theoretical approaches in guidance and career counseling consider “at risk work” as degraded work before it becomes degrading. The case of Mrs. V. is certainly not illustrative of every career issue. As a consequence, it cannot be generalized. Nevertheless, as a case study, it points out the main characteristics that make decent work a powerful concept to address today's challenges for research and practice in the field of career counseling. A main difficulty of today's world of work is that working conditions are deteriorating. Work has become increasingly precarious and constraining. This change does not only concern the poorest countries, where work may not even allow survival, but also rich ones, where work has become a form of alienation, even if it provides sufficient income. Thus, middle class populations are now concerned with the problem of decent work. This is the case for Mrs V. The purpose of this case study was mainly to highlight that decent work issues now concern all workers. It also points out the need for a clear and effective definition and framework. This notion allows us to focus on what makes a work situation disintegrated to the point that it is not acceptable, as framed by the principles of human rights.

The distinction is of great importance. Indeed, while degraded work can be upgraded, repaired, and prevented, indecent work needs to be fought with multiple political, economic, humanitarian, collective, and individual actions. A psychosocial definition of decent work raises the question of individual and socially acceptable limits of working conditions. While these limits can more easily be established on the basis of law or economics, individual acceptability is more difficult to define. For example, some constraints are legitimated by individuals even if they correspond to degrading working conditions (e.g., because of poverty, but also the neoliberal system that produces inequality to the point of questioning the individual attitudes of solidarity and democracy).

Thus, it may be easier to fight for decent work on a collective level than on an individual level. The main issue is indeed to understand how the conditions of decent work meet individual significance in order to allow or constrain an area of freedom, a space for action, and the capability for health. As underlined by Ricoeur, “suffering is not defined solely by physical pain, nor even by mental pain, but by the reduction, even the destruction, of the capacity for acting, of being-able-to-act, experienced as a violation of self-integrity” (Ricoeur, 1992, p. 190). Canguilhem's definition of health suggests a similar idea. “I feel good, because I feel myself capable of taking responsibility for my actions, of making things happen and of creating new connections among things that were not possible without me, but that wouldn't be the same without them. This is why I feel the need to learn what they are so that I can change them” (Canguilhem, 2002, p. 68, free translation). The starting point for this is therefore the subjective relationship to work, which can gradually be incorporated into collective and social issues. It follows the movement from the subjective to political dimensions through relationships with others. This movement primarily concerns the career counseling psychologist, whose action begins within the individual relationship, and continues through spiraling levels of collective action that promotes a progressive capability for self construction (a psychosocial activity of self-construction through work).

## Author contributions

The author confirms being the sole contributor of this work and approved it for publication.

### Conflict of interest statement

The author declares that the research was conducted in the absence of any commercial or financial relationships that could be construed as a potential conflict of interest. The reviewer MK and handling Editor declared their shared affiliation, and the handling Editor states that the process nevertheless met the standards of a fair and objective review.
